# Towards the routine use of *in silico* screenings for drug discovery using metabolic modelling

**DOI:** 10.1042/BST20190867

**Published:** 2020-05-05

**Authors:** Tamara Bintener, Maria Pires Pacheco, Thomas Sauter

**Affiliations:** Life Sciences Research Unit, University of Luxembourg, Esch-Alzette, Luxembourg

**Keywords:** cancer, drug repurposing, drug target discovery, metabolic modelling, personalized medicine, systems biology

## Abstract

Currently, the development of new effective drugs for cancer therapy is not only hindered by development costs, drug efficacy, and drug safety but also by the rapid occurrence of drug resistance in cancer. Hence, new tools are needed to study the underlying mechanisms in cancer. Here, we discuss the current use of metabolic modelling approaches to identify cancer-specific metabolism and find possible new drug targets and drugs for repurposing. Furthermore, we list valuable resources that are needed for the reconstruction of cancer-specific models by integrating various available datasets with genome-scale metabolic reconstructions using model-building algorithms. We also discuss how new drug targets can be determined by using gene essentiality analysis, an *in silico* method to predict essential genes in a given condition such as cancer and how synthetic lethality studies could greatly benefit cancer patients by suggesting drug combinations with reduced side effects.

## Introduction

Since Otto Warburg, it is known that some cancer cells have an altered metabolism such as preferring the production of ATP from aerobic glycolysis over oxidative phosphorylation [1]. What was believed to be the consequence of high mutation rates in cancer cells, is now regarded as required rewiring of metabolism, tailored by mutations and selection, to meet the high need for energy and cellular building blocks to sustain rapid proliferation rates [2]. This altered metabolism in cancer cells is an important research topic, as it potentially allows identifying cancer-specific vulnerabilities that could be targeted without harming healthy cells and hence are expected to have fewer side effects.

A wide catalogue of mutations across different tumours was gathered by the COSMIC database [3], as well as large transcriptomic datasets from thousands of cancer cell lines (CCLE [4], NCI-60 [5], 1000 Genomes Project [6,7]) and cancer patients such as TCGA [8] or Metabric [9]. The bottleneck in the understanding of metabolic rewiring is the integration of huge amounts of cancer data gathered from different experimental settings and literature. Genome-scale and context-specific models [10], that have been successfully used for the integration of -omics data, are very promising approaches that allow, among others, to understand how mutations affect cancer metabolism by mapping them onto context-specific models to study their metabolism [11] and to determine if the phenotype can be rescued by alternative pathways.

**Fig. 1.**
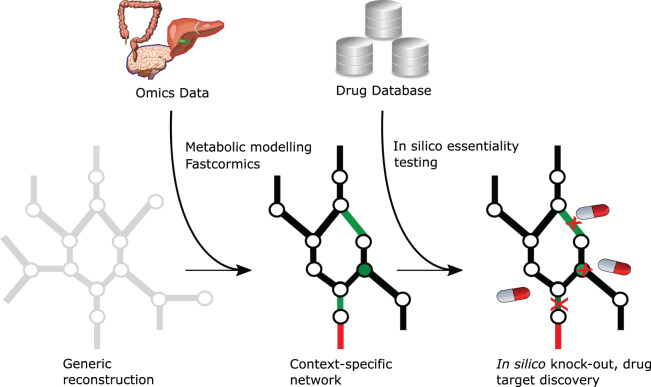
**Drug repurposing workflow using metabolic modelling and public databases.** A context-specific reconstruction (black network) can be extracted from a generic reconstruction (grey network) using -omics data and context-specific model reconstruction algorithms such as FASTCORMICS. Circles and lines represent metabolites and reactions, respectively. Genes that can be targeted by existing drugs and oncometabolites are mapped to the model to obtain a set of targetable reactions (green lines) or metabolites (green circles). To identify essential genes, reactions, or metabolites, one or more objective function(s) (red line) can be set and the effect of a drug-induced knockout on the objective function(s) can be simulated by preventing the targeted reactions to carry a flux. Depending on the network topology, the knockout can either have no effect on the flux through the objective function(s), or the flux is reduced if alternative pathways are present, or the knockout can cause a loss of all the flux through the objective function.

More interesting applications of genome-scale metabolic models are *in silico* knockout studies to discover cancer-specific essential genes [12] that could serve as potential drug targets or to identify oncometabolites by blocking the flux of the enzyme that consumes these metabolites [13]. A workflow using these approaches has previously been published [14] and is depicted in Figure 1. Because the *in vitro* identification of drug targets and drug screenings is a meticulous task, with drug combination screenings having endless possibilities, metabolic modelling can be used to narrow down the number of targets, therefore reducing the time and costs of experiments.

Furthermore, context-specific models can be used to identify cancer-specific flux distributions using random sampling [15,16], flux variability analysis [17], FBA [18], parsimonious FBA [19], or cancer-specific sub-pathway activation patterns by combining metabolic models with machine-learning approaches [14].

In this review article, we will discuss the current advances in metabolic modelling in regards to analysing cancer metabolic rewiring as well as possible applications in drug discovery.

## Cancer and metabolic modelling

### Metabolic alteration in cancer and their potential role as drug target

Oncogenes and tumour suppressor genes have, besides their iconic targets, metabolic targets that have been shown to act as metabolic regulators. For example, the constitutive expression of MYC affects glycolysis [20] and glutamine metabolism [21], whereas mutations or inactivation of the tumour protein p53 can lead to an increase in glycolysis while inhibiting gluconeogenesis [22]. Furthermore, mutations in the metabolic enzymes themselves can be a driving force for cancer such as mutations in the fumarate hydratase [23] and succinate dehydrogenase [24] have previously been associated with cancer by increasing cellular vascularization, invasion, and metastasis through the action of HIF-1α [25]. Loss-of-function mutations can cause the accumulation of fumarate and succinate, that are competitive inhibitors of α-ketoglutarate-dependent dioxygenases, perturbing histone and DNA demethylation [26]. Other enzymes (IDH1 and IDH2), when mutated, can also affect the activity of HIF-1α subunits by accumulating 2-hydroxyglutarate, a product of the conversion of α-ketoglutarate, mainly taking place in cancer cells [27].

Even though metabolic alterations play a lesser role in the contribution to cancer morphology and progression than mutations, these enzymes, whose deregulation causes the accumulation of sub-products of metabolism, are potential drug targets [28–31]. Other alterations with no transforming power themselves can facilitate the production of building blocks or maintain the redox state. These enabling alterations are often under the control of tumour suppressors or oncogenes and could be considered as potential drug targets.

### Metabolic models of cancer and context-specific models

Metabolic models are powerful tools to identify metabolic alterations and mechanisms in human diseases such as Alzheimer’s [32,33], to predict biomarkers for inborn errors of metabolism [34,35], liver metabolism [36,37], pathogen infection of alveolar macrophages [38], obesity [39], Leigh syndrome fibroblasts [40], diabetes [41], co-morbidity [42], obesity and diabetes application have been reviewed in [43] as well as drug target prediction in cancer [12,14,18,44–52].

Two different strategies are being used to study cancer metabolism:

The first approach, a bottom-up approach, aims to reconstruct a cancer core metabolic model, which only contains reactions present in all cancer samples. The small size of these models enable them to be manually curated and thoroughly analysed. For example, a model of ATP production showed that the Warburg effect is dependent on glucose uptake [53], a core model including the main metabolic pathway showed that the blackcancer phenotype induces metabolic changes [45], or the presence of metabolic differences in three cancer core models [18].

In the second approach, cancer genome-scale models are reconstructed using context-specific building algorithms such as MBA [37], iMAT [54], INIT [46,47], GIMME [55], PRIME [49], mCADRE [56], RegrEx [57], CORDA [58], FASTCORE family [14,59,60] using patient transcriptomic data as input. An overview of the model-building algorithms can be found in Supplementary Table S1. Data from various patients and samples can be pooled to reconstruct a single cancer type model or subgroup model of different patients. Even though pooling samples allows building models that are more robust to noise while displaying common alterations, the creation of patient-specific models without pooling allows detecting less common rewiring strategies [61].

Because metabolic rewiring strategies are tightly related to the identification of novel anticancer drugs, metabolic models were often used to identify potential drug targets.

The first genome-scale metabolic model of cancer was presented by [12] in order to study common metabolic alterations in cancer. The model is based on the manual selection of highly expressed core genes from the NCI-60 cancer cell lines as well as a minimal set of reactions needed to activate the core genes via an MBA. A total of 52 cytostatic metabolic drug targets were successfully predicted using *in silico* gene deletion and validated using sh-RNA screening data.

In a follow-up paper, [44] further investigated the effects of synthetic lethality in FH1-deficient cells, a deficiency that can lead to renal-cell cancer. The same model building approach was used as in [12] to create one deficient and one control model for FH1 that showed that the inhibition of Hmox is synthetically lethal in FH1 deficient cells.

More cancer-specific models shortly followed by integrating cancer data with different genome-scale reconstructions and model-building algorithms for data integration (see Supplementary Table S2) such as models for each of the cell lines in the NCI-60 to identify metabolic sub-pathways that provide energy and lipids for cancer growth [62] or HCC models that allow stratifying patients according to acetate utilization [63]

Recently, due to the decrease in computational demands and reconstruction times of context-specific metabolic models, initiated by the publication of FASTCORE [59] and due to the number of published cancer metabolic models, metabolic modelling could be combined with machine learning.

In a pioneer study, Christian Diener and colleagues [19] used regression approaches to predict cancer growth rates from the TCGA dataset while using the NCI-60 cancer cell line panel and TCGA as a training set. They showed that patients with a high predicted growth rate have a worse survival expectancy. Furthermore, they used the predicted growth rates to obtain the flux distributions via parsimonious FBA for more than 3000 samples using already published cancer models and identified pathways that are up-regulated in cancer such as the pentose phosphate pathway, retinol, branched-chain amino acid metabolism, and ROS detoxification.

In a second study, 10 005 context-specific models for the TCGA dataset were built using an extension of the FASTCORMICS workflow [60] for RNA-seq data [14]. A reverse feature selection approach was used to extract gene and reactions signatures that allow segregating between cancerous and control samples for 13 different cancer types. Furthermore, cancer models were shown to be smaller than their healthy counterparts and reactions from the cancer core metabolism were enriched for essential genes. Generic cancer-type models were also reconstructed to predict drug targets and propose drugs for repurposing in cancer. For colorectal cancer, three of the predicted drugs have been successfully validated *in vitro*.

### Personalized modelling and stratification of cancer patients

A future application of context-specific algorithms is the reconstruction and analysis of patient-specific metabolic models towards personalized treatment. This calls for algorithms that are robust to noise but nevertheless able to capture metabolic variations between different patients that result from inter-tumour variability. In some cancers, such as colon or breast cancer, numerous cancer subtypes were identified, each showing a different prognosis and drug response [64,65]. Being able to accurately model the inter-tumour heterogeneity would allow identifying subtype or even patient-specific drugs and biomarkers. The challenge resides in the distinction between real metabolic variations and noise or algorithm-related bias.

In recent years, benchmarking methods have been proposed to increase the quality of context-specific algorithms [66–68] and their generic reconstructions from which context-specific models are extracted from [69]. Standardizing the benchmarking workflows as well as eliminating any heuristic thresholds during the model reconstruction will improve the quality of the context-specific models so that they could eventually be used in personalised medicine.

Another hurdle that needs to be overcome is the intra-tumour heterogeneity. As tumours can be composed of different clones carrying different mutations, the reconstruction of models based on these biopsies might miss some of the clones and modellers risk to predict drugs that only select for clones that were captured by the biopsy. Furthermore, the use of bulk RNA-seq data might mask the intra-cellular variation. The next logical step would be to take biopsies at different locations of a tumour and to build single-cell models.

### *In silico* gene deletions are used to predict drug targets

The possibility to reconstruct context-specific models based on genomic and transcriptomic data allows for the exploration and comparison between the metabolism of different tissues, conditions, and patients. Thus, the metabolism of cancer cells can be compared with their healthy counterpart tissue (structural analysis) and alternative pathways can be elucidated. Furthermore, new potential drug targets with low toxicity can be predicted using *in silico* gene deletions or essentiality analysis [70] by focussing on cancer-specific vulnerabilities.

During gene essentiality analysis, the flux controlled by the knockout genes are set to zero (according to the gene–protein–reaction rules) and flux balance analysis [71] is run to determine the maximum flux through an objective function before and after the gene knockout [72]. In general, essential genes are defined as genes whose knockout affects the growth or survival of a cell, therefore, they are often used as a surrogate for potential drug targets. Conventionally, the objective function is defined as biomass production and often used to determine the growth rate of a cell [73]. This might be true for fast proliferating cells such as cancer but not for non-proliferating cells such as neurons. It is therefore important to choose the correct objective function (which differs between cell types, tissues and species) for the model [74] and to define a cancer and tissue-specific biomass instead of relying on the *Escherichia coli* biomass, currently used in most metabolic models. Even though one could take the ATP demand reaction as an objective for these cells, it would be more suitable to have a well-defined set of metabolic tasks that a cell needs to fulfil [46].

Similar to gene essentiality analysis, synthetic lethality analysis knocks down two genes simultaneously and the flux through the objective function is measured. Whereas the knockout of one gene might not have a significant effect on the cell, the knock-down of two genes can result in lethality or significantly reduced cell functioning. Synthetic lethality studies have already shown promising results in *E. coli* [72,75] and can be used in anticancer therapy [12,76]. Because cancer cells have high mutation rates and thus some genes are shut down *a priori*, synthetic lethality takes advantage of these non-lethal mutations in cancer to specifically kill malignant cells without harming healthy cells. Therefore, patients can also benefit from synthetic lethality studies because drug combinations that target multiple genes of a synergistic lethal couple are less likely to cause resistance as it is more difficult for cancer cells to simultaneously develop resistance to two targets [77]. Moreover, the use of drug combinations allows reducing the dosage which is in turn likely to reduce the toxicity of each compound [78–80].

Several algorithms can perform single, double, and multiple knockouts on genes as well as on reactions and metabolites to simulate the effect of oncometabolites (Table 1). Oncometabolites are competitors for the access to the catalytic site of an enzyme, therefore inhibiting the normal conversion of a metabolite. Reactions consuming these metabolites are regarded as inactive during the simulations. Besides the usual brute force approaches, several algorithms were proposed that used a more targeted approach to reduce computational demands. Notably, an algorithm for the study of synergistic lethality was proposed that allows impairing undesired functions while guaranteeing the production of key metabolites [81].

Another strategy to identify synergistic lethality has been proposed in 2017 and is based on genetic minimal cut sets or gMCSs [82]. Their framework finds the minimal number of genes that have to be knocked out in order to block a metabolic task such as the biomass production. The analysis is performed on the generic reconstruction to avoid any bias linked to heuristic thresholds in the -omics data during the context-specific model reconstruction. The -omics data is only used to drive the selection.

**Table 1.** Knockout tools

Deletion type	Tools and algorithms
Single gene deletion	singleGeneDeletion of the Cobra toolbox [83]) (Flux Balance Analysis, MOMA, linear MOMA)
	Fast-SL [84]
	FastMM_singleGeneKO_multi [85] (Flux Balance Analysis)
Double gene deletion	doubleGeneDeletion of the Cobra toolbox (Flux Balance Analysis, MOMA, linear MOMA)
	Fast-SL
	FastMM_doubleGeneKO_multi (Flux Balance Analysis)
	gMCSs [82]
	OptKnock [86]
Multiple gene deletion	Fast-SL
	gMCSs [82]
	OptKnock
Single reaction deletion	singleRxnDeletion of the Cobra toolbox (Flux Balance Analysis, MOMA, linear MOMA)
Single metabolite deletion	singleMetKO from fastMM toolbox
Double metabolite deletion	doubleMetKO, from the fastMM toolbox

Consequently, *in silico* knockouts are promising to find drug targets in cancer as has already been demonstrated in several publications [12,14,44–49,87]. Notably, [87] and [14] performed *in silico* drug predictions and found Ifenprodil as a potential repurposed drug for the prostate cancer and Naftifine, Mimosine and Ketoconazole for colon cancer, respectively. Both groups validated their respective targets *in vitro*.

However, robust validation methods need to be established for the predicted drug targets. One possibility is to compare the predictions to an essential gene screenings [88]. Even though there exist different high-throughput screenings that used shRNA [89], RNAi [90], or CRISPR/Cas9 on cell lines [91–93] and patient-derived glioblastoma cells [94] to experimentally determine essential genes, their application is still limited: Screenings cannot be performed for every condition and cell type and they only allow targeting one gene at the time. Thus, complete (synthetic) lethality screenings for all cancer and cell types targeting two genes at the same time would be practically impossible due to the sheer number of possibilities.

However, the Cancer Dependency Map Project made an effort to gather information about gene and drug screenings while combining the data in a comprehensive and regularly updated website (https://depmap.org). The aim of this project is to characterize as many cell lines as possible and identify potential genetic vulnerabilities and drug targets in cancer. The Cancer Dependency Map was created by combining genetics screens, cell line characterization data and drug sensitivity data from Achilles [89,95], DRIVE [96], Score [97], CCLE [4], CCLF [98], PRISM [99], CTRP [100–102], GDSC [103], and CTD2 [104].

## From potential candidate gene to drug target validation

Even though the process of finding appropriate drugs for candidate genes is straightforward, some pitfalls will need to be overcome. This can be achieved by using the databases and tools described in the following section.

### Challenges

The first challenge is the inconsistency of nomenclature used by the creators of metabolic models and the second is database updates that might significantly alter results between two releases.

#### Nomenclature

In the metabolic modelling community, there exist different genome-scale metabolic reconstructions for humans (and other organisms) that can in themselves already be seen as a primary database [105–112]. A genome-scale metabolic reconstruction is a collection of all the known genes, reactions, metabolites, and their interactions that are present or can take place in any given cell at the time of reconstruction.

First off, there is no consensus for the identifiers that should be used in a reconstruction. For example, Recon 1 [105] and Recon 2 [108] use Entrez Gene identifiers [113], whereas HMR [110] and Recon 2.2 [111] use Ensembl gene identifiers [114] and HGNC identifiers [115], respectively. The same goes for metabolite identifiers which can be in the BiGG [116], SEED [117], or BioCyc [118] format, but might also have more common identifiers such as the CAS number, ChEBI ID, PubChem ID, or KEGG ID associated. In some versions of some reconstruction, there is sometimes a mix of different identifiers, which makes matching identifiers between the models difficult. Similar problems arise with the names of the proteins, interacting drugs, chemicals, and diseases as many databases associate internal identifiers to them.

The problem of a non-standardized vocabulary is well known in the scientific community [119] and makes data retrieval and integration challenging [120].

#### Data retrieval and update intervals

Even though many databases offer online tools to the user that are useful for looking up a few genes or drugs but with big data appearing, more holistic approaches are being used and the user wants a whole overview of the data. Unfortunately, not all databases offer direct and free access to downloadable files, making data retrieval unnecessarily difficult.

Another challenge with online databases is maintenance and update intervals. Some databases have scheduled updates, which is *per se* good practice, but it also requires re-downloading, updating, or adjusting a user-defined script. With updates, besides the addition of content, some entries in a database might be changes or be withdrawn such as for some genes. Unfortunately, other databases are not updated on a regular basis and they are left with outdated information that needs to be revised. It is therefore important to mark the version number of a database.

### Resources and databases

In general, for drug target prediction in cancer (and other diseases), the most important databases are interaction databases, which link a gene or mutation to a specific disease, or a drug to a protein. Interaction databases are vital to interconnect the different pieces of information and to create a more global and focussed view on the disease and its treatment strategies.

In their publication, [121], described different approaches for data integration at a systems level and some of the available data repositories. In the following subsection, we will shortly describe the most important databases for gene information, proteins, interactions and simply list others for further information. More information can also be found in the review from [122] that describe drug-related data types, and web-based drug repositioning tools.

#### Gene databases

After retrieving a list of essential genes, more information about these genes needs to be gathered.

The best-known databases for gene information are Ensembl [114], NCBI Entrez Gene [113], and HGNC [115]. It is useful to download a text file to convert the different identifiers (http://www.genenames.org/cgi-bin/download), Biomart (https://www.ensembl.org/biomart/martview/), David (https://david.ncifcrf.gov/conversion.jsp), or bioDBnet (https://biodbnet-abcc.ncifcrf.gov/db/db2db.php) to give some examples. If one is working with microarray data, the probe IDs should also be converted to the correct genes by downloading the gene annotation files for the used platform (https://www.ncbi.nlm.nih.gov/ for example).

Whereas some databases focus more on the expression of genes across specific tissues or conditions, such as the Human Protein Atlas [123] or CCLE [4], other databases collect information about gene mutations and their associated diseases such as ClinVar [124] or COSMIC [125], which can be useful tools for model validation.

A brief overview of these databases can be found in the Supplementary Tables S3, 43, and S5.

##### Essential gene screenings

To this date, there exist several large-scale collections of essential gene screenings for human and cancer cell lines that can be used to validate predicted essential genes (Supplementary Table S6). As different methods are used to determine essential genes in a given cell line or tissue, the results are not always comparable and finding a core set of essential genes in cancer is still ongoing. The Cancer Dependency Map (https://depmap.org) is currently gathering and harmonizing several essential gene screenings into one comprehensible platform.

As stated in the beginning, gene essentiality analysis is often performed in metabolic modelling studies in order to predict essential genes that could be considered as drug targets. Here, *in vitro* performed essential gene screenings can be used to validate the predicted essential genes using statistical test, e.g. a hypergeometric test. This is especially useful to predict drug targets because one could directly assess the effect of the gene deletion in a cancer cell compared with a healthy cell.

#### Proteins, drug targets, and protein-drug interactions

Besides genes, protein databases have become increasingly important as they include information on the protein sequence, structure, and biological function(s), which are relevant for drug target prediction and validation.

The first available protein database was The Protein Data Bank [126] (https://www.wwpdb.org/), which currently stores more than 150 000 entries on the protein structure. Whereas some databases, such as PDB, focus more on the 3D structure of a protein, other databases such as UniProt [127] focus more on the sequence of a protein. These can be useful to determine new drug binding sites. There also exist databases that focus more on the biological functions and pathways of a protein such as Gene Ontology [128] and KEGG [129]. Extensive lists on protein databases with their advantages and drawbacks have already been discussed elsewhere [130], here we will focus more on protein interaction databases for drug discovery.

By linking the predicted essential genes with their respective proteins, protein interaction or protein binding databases can be used to retrieve a list of known drugs or chemicals that interact with these proteins/genes. Examples of such databases are the Binding Database [131], which mainly gives information on the binding affinity between a protein and a ligand but also pharmacokinetics, 3D structures, and links to other databases, the DrugBank [132], which focusses more on the drugs themselves and its pharmacokinetics but also provides information on the protein targets and the type of interaction (i.e. inhibitor, activator, substrate,…) and the Stitch [133] database, which is a collection of chemical and protein interaction networks with biological evidence that also focuses on the interactions between chemicals. There also exist other protein and drug interaction databases which are listed in Table 2.

**Table 2.** Drug and interaction databases

Name	Description	URL	Citation
BindingDB	Protein binding database	https://www.bindingdb.org	[134]
CancerDR: Cancer Drug Resistance Database	Collection of 148 anticancer drugs, their targets and effectiveness	http://crdd.osdd.net/raghava/cancerdr/	[135]
CancerResource	Drug-target interactions in cancer	http://data-analysis.charite.de/care/	[136]
CGP: Cancer Genome Project	Screening of cancer cell lines with drug response data (now included in COSMIC)	http://www.sanger.ac.uk/genetics/CGP/CellLines/	[137]
ChEMBL	Drug bioactivity data	https://www.ebi.ac.uk/chembl/	[138]
Connectivity Map	Drug screenings	https://clue.io/	[139]
CTD: Comparative Toxicogenomics Database	Gene-Drug-Disease interactions	http://ctdbase.org/	[140]
CTRP: Cancer Therapeutics Response Portal	Drug Sensitivity in Cancer, 860 cell lines and 481 compounds	https://portals.broadinstitute.org/ctrp/	[100]
DGIdb: The Drug Gene Interaction Database	Gene-Drug interactions	http://dgidb.genome.wustl.edu/	[141]
DrugBank	Gene-Drug interactions and drug information	https://www.drugbank.ca/	[142]
gCSI: The Genentech Cell Line Screening Initiative	Independent screening of 410 cancer cell lines to 16 agents of CCLE and GDSC data	http://research-pub.gene.com/gCSI-cellline-data/	[143]
GDSC: Genomics of Drug Sensitivity in Cancer	Drug response data and drug sensitivity in cancer	https://www.cancerrxgene.org/	[103]
Growth rate inhibition metrics	Dose-response data for breast cancer (from LINCS)	http://www.grcalculator.org/grtutorial/Home.html	
GSK: GlaxoSmithKline cell line collection	Response profiles of 19 compounds in 311 cell lines		[144]
Hetionet	Combination of 29 public databases on genes, disease, drugs, side effects,…	https://het.io/	[145]
IDG: Illuminating the Druggable Genome	Drug-targeted protein families	https://druggablegenome.net/	[146]
Kegg Drug	Information on drugs and their targets	https://www.genome.jp/kegg/drug/	[129]
LINCS: Library of Integrated Network-Based Cellular Signatures	Gene expression and drugs	http://www.lincsproject.org/	[147]
NPC: NCGC Pharmaceutical Collection	Drug screening data &	https://tripod.nih.gov/npc/	[148]
Orphanet	Rare diseases and orphan drugs	http://www.orpha.net	[149]
Pharmacodb	Collection of anticancer drug screenings	http://pharmacodb.ca/	[150]
Pharos	Knowledgebase for the druggable genome	https://pharos.nih.gov/idg/index	[151]
PubChem	Chemical database	https://pubchem.ncbi.nlm.nih.gov/	[152]
repoDB	Clinical trial and repositioning database	http://apps.chiragjpgroup.org/repoDB/	[153]
SIDER: Side Effect Resource	Side effect database for drugs	http://sideeffects.embl.de/	[154]
STITCH	Drug Target Discovery	http://stitch.embl.de/	[133]
SuperTarget	Drug targets, side effects	http://insilico.charite.de/supertarget/	[155]
T3DB	Gene-toxin database	http://www.t3db.ca/	[156]
TCM Database	*in silico* drug screenings of Traditional Chinese medicine	http://tcm.cmu.edu.tw/	[157]
The Drug Repurposing Hub	Drug repurposing	https://clue.io/repurposing	[158]
Transformer (former SuperCYP)	Cytochrome-drug interactions	http://bioinformatics.charite.de/transformer/	[159]
TTD: Therapeutic Target Database	Drug targets	http://bidd.nus.edu.sg/group/cjttd/	[160]
UniProt	Protein database	www.uniprot.org/	[127]
YaTCM	Linking traditional Chinese medicine to targets and diseases	http://cadd.pharmacy.nankai.edu.cn/yatcm/home	[161]

##### Drugs and side effects

Not only the development of new drugs is greatly hampered by drug efficacy and safety [162], severe side effects are responsible for fails during clinical trials, therefore, minimizing the toxicity of the drugs is necessary. Chemotherapeutic agents, for example, target proteins that are present in all rapidly proliferating cells, cancer cells as well as healthy cells causing the side effect. Targeted cancer drugs, on the other hand, are more selective but also come with side effects and are not always able to eradicate all cancer cells due to cancer heterogeneity. The appearance of side effects of these more targeted drugs can be explained by the drug’s affinity for similar binding sites on another protein also called off-targets [163].

Moreover, using available data on drugs and their interactions, side effects of a drug have already been predicted solely based on *in silico* modelling [164–166]. By combining metabolic modelling with a drug repurposing workflow, the risk of severe side effects can be reduced by suggesting a combination of two or more lower dosed drugs than one single highly dosed drug based on the metabolic modelling results. For example, one could find drug synergies between currently used anti-cancer drugs and other drugs that might allow lowering the dose of the anti-cancer drug.

#### Databases and datasets for cancer

There exist many different datasets of varying sizes, quality, and research focus that are stored on platforms and repositories such as NCBI Gene Expression Omnibus (GEO) [167] and ArrayExpress [168] but more specific datasets such as The Cancer Genome Atlas with more than 11000 patient samples across 33 different tumour types [8], NCI-60 [5], 1000 Genomes Project [6,7] or the Cancer Cell Line Encyclopedia [4] also exist. For a more exhaustive list of cancer datasets, see Supplementary Table S7.

## Discussion

To study metabolic rewiring, two different strategies were adopted: the first focuses mostly on manually curated models of the core metabolism that were built from scratch or were extracted via model-building algorithms from already published models that were then extensively curated. The first strategy is very time consuming and only permits to capture more generic rewiring strategies. The second strategy takes advantage of the capacity of model building algorithms to reconstruct a large number of context-specific models to perform statistically relevant analysis. Whereas this approach is more subjected to noise and algorithm-related bias, it enables to capture metabolic rewiring strategies in different samples, tissues, cancers, and sub-populations.

Although context-specific metabolic models were successfully used to integrate patient data, their application to study tumour samples is still dependent of the accuracy of the model-building algorithm, the quality of the input reconstruction and the discretization/integration function used [66,67]. If the algorithm is too conservative, it will wrongly exclude lowly expressed reactions, mark alternative pathways as inactive and therefore overestimate the number of essential genes. The inverse is equally true, a model that falsely calls alternative pathways as active due to relaxed thresholds will underestimate the number of essential genes. Even though the remaining process of finding a matching drug in a database for a predicted candidate gene is straightforward, huge improvements can still be done on the modelling side, notably of the biomass composition. Currently, a very reduced number of biomass functions is published that are often used regardless of the tissue type and proliferation speed. The prediction power of a model could be improved by using more adapted biomass formulation, which considers fast and slowly proliferating cells.

Furthermore, the reaction to a drug can vary drastically from one patient to another where one might not be responding at all and another will suffer adverse effects. Therefore, patient stratification and tailored drug treatments are going to be a major challenge to find the most efficient drug or drug combination with the least side effects.

Here, metabolic modelling could be applied to predict patient-specific groups by using classifiers such as biomarkers or gene signatures that allow assigning patients into different metabolic groups or by using the metabolic variation captured by the metabolic models. These models could then be used to predict personalized drug targets and, eventually, treatments. Another important aim is to find drug combinations, which allow us to lower the overall dose and consequently reducing drug toxicity. But more importantly, cancer cells are less likely to simultaneously develop resistance to two (or more) different drugs and therefore drug combination treatments could show a higher success rate in killing cancer cells. Even though double and triple knockouts are possible, they cannot be performed experimentally for all drug combinations. As the development of a new drug is very risky and time-consuming, proposing drugs for repurposing using metabolic modelling could drastically help to develop new treatments.

## Perspectives

- **Highlight the importance of the field**: In this review, we describe a roadmap about how metabolic modelling can be used for drug discovery. We also cite the most important resources (databases and datasets) that can be used to determine novel drug targets and drugs.
- **A summary of the current thinking**: Current cancer therapies often fail due to the appearance of resistance inside the tumour. Metabolic rewiring is a known hallmark of cancer, thus using metabolic modelling can be used to identify cancer-specific vulnerabilities and predict novel drug targets.
- **A comment on future directions**: For the future, using personalized medicine, the creation of patient-specific models that capture inter-tumour heterogeneity as well as single-cell RNA-seq model that capture intra-tumour heterogeneity will greatly improve the drug response of a patient by increasing the effectiveness of a drug and reducing its side effect.
